# Biomarkers In Prediction of Acute Mesenteric Ischaemia: a prospective multicentre study (BIPAMI study): a study protocol

**DOI:** 10.1186/s12893-024-02491-3

**Published:** 2024-07-03

**Authors:** Kadri Tamme, Stefan Acosta, Alan Biloslavo, Martin Björck, Dumitru Casian, Dimitrios Damaskos, Alastair Forbes, Karri Kase, Kalle Kisand, Ines Lakbar, Vladislav Mihnovitš, Marko Murruste, Merli Mändul, Alexandre Nuzzo, Martin Padar, Joel Starkopf, Diego Visconti, Annika Reintam Blaser

**Affiliations:** 1 Institute of Clinical Medicine, University of Tartu, Tartu, Estonia; 2 Tartu University Hospital, Tartu, Estonia; 3 Department of Clinical Sciences, Lund University, Malmö, Sweden; 4 General Surgery Department, Cattinara University Hospital, Trieste, Italy; 5 Department of Surgical Sciences, Vascular Surgery, Uppsala University, Uppsala, Sweden; 6 Department of General Surgery Nr. 3, “Nicolae Testemitanu” State University of Medicine and Pharmacy, Chisinau, Moldova; 7 Vascular Surgery Clinic, Institute of Emergency Medicine, Chisinau, Moldova; 8 Department of General Surgery, Royal Infirmary of Edinburgh, Edinburgh, UK; 9 Anesthesia and Critical Care Department B, Saint Eloi Teaching Hospital, University of Montpellier, Montpellier, France; 10 Institute of Mathematics and Statistics, University of Tartu, Tartu, Estonia; 11 Estonian Genome Center, Institute of Genomics, University of Tartu, Tartu, Estonia; 12 Department of Gastroenterology, IBD and Intestinal Failure, Intestinal Stroke Center, AP-HP. Nord, Beaujon Hospital, Paris Cité University, Paris, France; 13 AOU Cittá Della Salute E Della Scienza, Turin, Italy; 14 Department of Intensive Care Medicine, Lucerne Cantonal Hospital, Lucerne, Switzerland

**Keywords:** Acute mesenteric ischaemia, Biomarkers, Diagnosis

## Abstract

**Background:**

Acute mesenteric ischaemia (AMI) is a life-threatening disease where early diagnosis is critical to avoid morbidity and mortality from extensive irreversible bowel necrosis. Appropriate prediction of presence of bowel necrosis is currently not available but would help to choose the optimal method of treatment.

The study aims to identify combinations of biomarkers that can reliably identify AMI and distinguish between potentially reversible and irreversible bowel ischaemia.

**Methods:**

This is a prospective multicentre study. Adult patients with clinical suspicion of AMI (*n* = 250) will be included. Blood will be sampled on admission, at and after interventions, or during the first 48 h of suspicion of AMI if no intervention undertaken. Samples will be collected and the following serum or plasma biomarkers measured at Tartu University Hospital laboratory: intestinal fatty acid-binding protein (I-FABP), alpha-glutathione S-transferase (Alpha- GST), interleukin 6 (IL-6), procalcitonin (PCT), ischaemia-modified albumin (IMA), D-lactate, D-dimer, signal peptide-CUB-EGF domain-containing protein 1 (SCUBE-1) and lipopolysaccharide-binding protein (LBP). Additionally, more common laboratory markers will be measured in routine clinical practice at study sites.

Diagnosis of AMI will be confirmed by computed tomography angiography, surgery, endoscopy or autopsy.

Student’s t or Wilcoxon rank tests will be used for comparisons between transmural vs. suspected (but not confirmed) AMI (comparison A), confirmed AMI of any stage vs suspected AMI (comparison B) and non-transmural AMI vs transmural AMI (comparison C). Optimal cut-off values for each comparison will be identified based on the AUROC analysis and likelihood ratios calculated. Positive likelihood ratio > 10 (> 5) and negative likelihood ratio < 0.1 (< 0.2) indicate high (moderate) diagnostic accuracy, respectively. All biomarkers with at least moderate accuracy will be entered as binary covariates (using the best cutoffs) into the multivariable stepwise regression analysis to identify the best combination of biomarkers for all comparisons separately. The best models for each comparison will be used to construct a practical score to distinguish between no AMI, non-transmural AMI and transmural AMI.

**Discussion:**

As a result of this study, we aim to propose a score including set of biomarkers that can be used for diagnosis and decision-making in patients with suspected AMI.

**Trial registration:**

NCT06212921 (Registration Date 19–01-2024).

**Supplementary Information:**

The online version contains supplementary material available at 10.1186/s12893-024-02491-3.

## Background

Acute mesenteric ischaemia (AMI) is a rare disease, difficult to diagnose due to lack of specific symptoms and reliable biomarkers [1]. Early diagnosis is crucial to avoid extensive irreversible bowel injury resulting in severe disability due to short bowel syndrome, or death. Despite availability of computed tomography (CT), vascular surgery and interventional radiology the mortality of AMI remains very high [2, 3]. While CT angiography is the gold standard of diagnosis of occlusive AMI, non-occlusive AMI (NOMI) has less distinctive CT signs [1]. Neither clinical symptoms nor CT scan perform well in any subtype of AMI in predicting whether bowel ischaemia is transmural or not, while this assessment is extremely important for choosing treatment method (endovascular revascularization vs open surgical approach).

A recent systematic review revealed no biomarker with high accuracy in diagnosis of AMI [4]. Several shortcomings in the existing literature were identified in this review: (1) Patients with strangulating bowel obstruction were included in most of the studies and the severity of ischaemia was seldom specified; (2) Biomarkers were commonly measured only once, with the measurement time point falling in different periods after occurrence of symptoms; (3) Combinations of biomarkers were rarely assessed; (4) Severity of bowel injury (non-transmural vs. transmural ischaemia) was not reported in many studies; (5) Reperfusion was not considered in any of the studies. Accordingly, we believe that biomarkers related to those sub-types have not been sufficiently studied, giving a rationale for this study.

There are obvious differences in pathophysiological mechanisms between different sub-types of AMI, whereas differences and similarities regarding biomarkers have not been studied.

Serum inflammatory markers interleukin-6 (IL-6) and procalcitonin (PCT), ischaemia modified albumin (IMA) and alpha-glutathione S transferase (alpha-GST) have shown relatively high predictive values for diagnosis of any type (transmural or not transmural) of AMI in humans [4]. Of coagulation factors, D-dimers performed moderately well in predicting transmural AMI in humans [4], while signal peptide-CUB-EGF domain-containing protein 1 (SCUBE-1) predicted early mesenteric ischaemia in animal experiments [5, 6]. The enterocyte injury marker intestinal fatty acid-binding protein (I-FABP) has been extensively studied in humans and has shown moderate diagnostic accuracy for diagnosis of AMI [4]. While D-lactate performed relatively poorly as a biomarker for AMI in humans [4], animal studies have shown promising results [7–9], also including intestinal barrier dysfunction markers like D-lactate and lipopolysaccharide binding protein (LBP) in potential combinations may lead to improved diagnostic accuracy.

Numerous animal studies have shown potential of different biomarkers in diagnosis of AMI but results in animal models with clear-cut onset are not easy to transfer to humans [5–11].

Non-occlusive mesenteric ischaemia (NOMI) and mesenteric venous thrombosis are difficult to study in animal models. Moreover, observation period after experimental AMI is usually short, and ischaemia and reperfusion periods commonly not distinguished. Human studies considering time of ischaemia and subsequent severity of intestinal injury are lacking, as well as studies assessing biomarker dynamics during reperfusion after successful revascularisation.

Different biomarker combinations may be optimal for different sub-types and severity of AMI, and for different time points of measurement after onset of symptoms.

The current study will be undertaken to identify combinations of biomarkers that can reliably identify AMI when compared to suspected but not confirmed AMI, ideally allowing to distinguish between non-transmural and transmural bowel damage. We aim to propose a practical score for clinical decision-making.

## Methods

### Objectives

#### Primary objective:

- To identify a combination of biomarkers that distinguishes patients with transmural AMI from patients with no AMI with a positive likelihood ratio of > 10 and negative likelihood ratio of < 0.1 at least at one measurement time point before any treatment of AMI (comparison A).

#### Secondary objectives:

- To identify a combination of biomarkers that distinguishes patients with AMI (any stage—non-transmural or transmural necrosis) from patients with no AMI with positive likelihood ratio of > 10 and negative likelihood ratio < 0.1 at least at one measurement time point before any treatment of AMI (comparison B).
- To identify combination of biomarkers that distinguishes patients with non-transmural AMI from patients with transmural AMI with positive likelihood ratio of > 5 and negative likelihood ratio < 0.2 at at least one measurement time point before any treatment of AMI (comparison C).

#### Tertiary objectives:

- To assess performance of identified biomarker combinations in different subtypes of AMI (arterial occlusive, NOMI, mesenteric venous thrombosis)
- To describe patterns of individual biomarkers before and after treatment of AMI, separating between ongoing ischaemia and reperfusion.

### Study design and eligibility criteria

This is a prospective multicentre study.

All adult patients with clinical suspicion of acute mesenteric ischaemia will be considered eligible for the study.

#### Inclusion criteria

Age 18 years or older.

Initial decision in favour of further diagnostics of mesenteric ischaemia.

#### Exclusion criteria

Age < 18 years.

Consent declined or withdrawn by patient or next of kin.

Chronic mesenteric ischaemia without an acute event.

Immediate decision for withdrawal of further diagnostic workup and active treatment.

Referral from another hospital more than 8 h after diagnosis of AMI.

Strangulated bowel obstruction (SBO) as a primary verified diagnosis.

AMI incidentally diagnosed at surgery without previously having been considered.

### Definitions

#### Suspicion of AMI will be defined as:

1. Clinical suspicion of AMI. Factors indicating AMI are (not limited to): abdominal pain (usually diffuse and strong) usually supported with appropriate phenotype for AMI (older age, atrial fibrillation) and absence of an obvious alternative diagnosis after primary clinical assessment.
2. Clinical suspicion of mesenteric venous thrombosis. Unspecific and less intense abdominal pain, risk factors for venous thrombosis (mainly thrombophilia and obesity).
3. Clinical suspicion of NOMI (distended abdomen, ileus or unexplained worsening of shock) in critically ill patients with hypoperfusion and/or hypotension, often receiving vasopressors.

#### Confirmed AMI will be defined as:

1. Total or subtotal occlusion of any large mesenteric vessel visualized on CT, magnetic resonance imaging (MRI) or plain angiography with acute symptoms (e.g. abdominal pain, diarrhoea, shock, mucosal sloughing).
  - Imaging finding without any symptoms of intestinal ischaemia and without any laboratory abnormalities is not sufficient to confirm AMI (probably indicating chronic mesenteric ischaemia). For definitive confirmation in such case either endoscopic finding of mucosal ischaemia, surgical finding of transmural ischaemia leading to resection or palliation, or autopsy finding of any stage of bowel ischaemia is needed to confirm the diagnosis.
2. Non-occlusive mesenteric ischaemia confirmed by either endoscopy, surgery or autopsy. A suspicion of NOMI in CT, MRI or angiography without being confirmed by any of these methods remains a suspicion of AMI.

Each case without definitive confirmation of AMI or without definitive confirmation of the subtype of AMI will be carefully reviewed by steering committee members and discussed with respective site to confirm the final categorization.

Patients with strangulating bowel obstruction (SBO) as an immediate verified diagnosis at the time of the first clinical assessment will be excluded from this study. If a SBO was identified as a cause of AMI only after including the patient in the study (as suspected AMI), this patient should continue in the study.

Patients with acute-on-chronic mesenteric ischaemia will be included.

#### Subtypes of AMI assessed in subgroup analyses will be:

1. Occlusive arterial AMI
  - Thrombosis
  - Embolism
  - Unclear
2. Mesenteric venous thrombosis
3. NOMI

Patients with suspicion of AMI will be identified by treating physicians who inform study investigators. Study inclusion, confirmation and subtype of AMI will be validated by at least one study investigator. Principal investigator at each site is responsible for building up the study team and logistics as best suitable for local conditions.

In case the mechanism of AMI remains unclear or there is another specific mechanism (e.g. dissection, aneurysm, abdominal compartment syndrome), this case will be discussed in steering committee and allocated to the most suitable category and mentioned respectively in the final report.

### Study period and sites

We will invite sites participating in the AMESI study [3] and in the GUTPHOS study (NCT05909722) and open the study for other interested sites, while limiting participation to sites located within a distance range from Tartu, Estonia that allows safe shipment of frozen samples. There is no limitation for the category of the hospital, but we aim to recruit acute care hospitals commonly encountering at least one patient with AMI per month. Each site is expected to include at least 8 patients (in total for suspected and confirmed AMI) within the first 4 months of the study, until the interim analysis, and at least 20 patients in total. The study will start in October 2024 the earliest, with planned recruitment for 8–10 months, whereas the total length of the study period will be defined after the interim analysis at four months after the study start.

### Study procedures

Decisions regarding diagnostics and treatment in study patients will not be influenced by the study, the only study procedure is additional blood sampling.

#### List of biomarkers

The following biomarkers potentially identifying AMI will be centrally measured in Tartu, Estonia: intestinal fatty acid-binding protein (I-FABP), alpha-glutathione S-transferase (Alpha- GST), interleukin 6 (IL-6; LOINC# 26881–3), procalcitonin (PCT; LOINC# 75241–0), ischaemia-modified albumin (IMA; LOINC# 75239–4), D-lactate (LOINC# 14045–9), signal peptide-CUB-EGF domain-containing protein 1 (SCUBE-1), lipopolysaccaharide-binding protein (LBP; LOINC# 88054–2), D-dimers (LOINC# 48065–7). Logical Observation Identifiers Names and Codes (LOINC) is a universal standard database for identifying medical laboratory observations [12].

The list will be kept open for late changes if any novel biomarker emerges before the first laboratory analyses are performed, provided that this marker is possible to measure without an additional sampling tube.

Additionally to centrally measured biomarkers the sites will be asked to measure the following at local hospital laboratories:

- Blood lactate (arterial, where available), pH, bicarbonate and base excess at each of the planned time points as appropriate for each particular patient.
- Creatinine (LOINC # 14682–9), high sensitive Troponin T (LOINC # 89576–3) or Troponin I (LOINC # 89577–1), white blood cell count (WBC, LOINC # 6690–2) and C-reactive protein (CRP, LOINC# 1988–5) at time points 1, 2 and 6.

#### Blood sampling time points

Two sampling tubes (one serum and one plasma, arterial or venous) will be collected at each measurement point, allowing central measurement of aforementioned biomarkers.

**Fig. 1:**
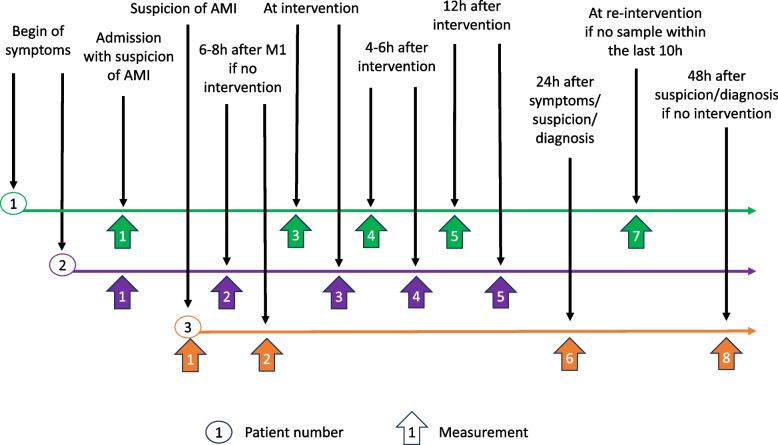
Different scenarios as examples. Measurement numbers are based on the list above. Legend: Patient 1 has a treatment intervention within 6–8 h after hospital admission due to AMI. Blood sampling points will be on admission (M1), at the intervention (M3), 4–6 h after the intervention (M4), 12 h after the intervention (M5) and at reintervention (M7; if reintervention occurs within 72 h). Patient 2 has a treatment intervention later than 6–8 h after admission due to AMI. Blood sampling points will be on admission (M1), 6–8 h after M1 (M2), at the intervention (M3), 4–6 h after the intervention (M4) and 12 h after the intervention (M5). There was no re-intervention within 72 h. Patient 3 has suspicion of AMI while hospitalized and has no treatment intervention. Blood sampling points will be on suspicion of AMI (M1), 6–8 h after M1 (M2), 24 h after suspicion or diagnosis of AMI (M6) and 48 h after suspicion or diagnosis of AMI (M8)

Depending on whether AMI is confirmed or not, and on treatment methods applied, different measurement time points will apply for each patient (Table 1 and Fig. 1).

**Table 1:** Sampling time points for biomarkers measurements (M)

M no	Time point for blood sampling	Confirmed AMI with intervention	Suspicion of AMI*	Confirmed AMI without intervention
M1	Admission (suspicion, if in-hospital)	x	x	x
M2	6-8 h after the first sample if no intervention performed by then	x (if no intervention before 6-8 h)	x	x
M3	At intervention	x (incl. i/a vasodilation)		
M4	4–6 h after intervention	x		
M5	12 h after intervention	x		
M6	24 h after the first sample if no intervention		x (if suspicion still actual)	x
M7	At the time of re-intervention	x (if any within 72 h)		
M8	48 h after the first sample		x (if suspicion still actual)	x (if no intervention)

* If the case is confirmed, the patient will be moved to either column “Confirmed AMI with intervention” or “Confirmed AMI without intervention”

#### Handling of blood samples and details of laboratory analytics

One lithium-heparin plasma tube (LH-tube) and one serum clot activator/gel tube (CA- tube) will be collected at each measurement point. Tubes will be centrifuged (10 min at 2000 g) no more than 2 h after blood collection and separated plasma/serum will be aliquoted to storage tubes. The samples will be stored at study sites at -80 °C and sent to the central laboratory in Tartu in two batches: one batch of samples at 4 months after the study start and the second batch after the end of the study.

Specific methods for biomarkers measurements are presented in Table 2.

**Table 2:** Measurement methods of biomarkers

Biomarker	Rationale	Measurement	Data
**Enterocyte damage marker**			
I-FABP	Produced in small intestine (and colon?)	Plasma: LH-tube; Amount: 0.1–0.2 mL; Method: ELISA—Human FABP2/I-FABP Quantikine (DFBP20) Provider: R&D Systems (USA)	Moderate accuracy in humans (4); not IVD-R compatible
**Intestinal barrier dysfunction markers**			
D-lactate	Produced by intestinal microflora	Plasma: LH-tube; Amount: 0.2 mL Method: colorimetric—D-Lactate Assay Kit (MAK336) Provider: Sigma Aldrich (Germany)	Low accuracy in humans (4). Animal studies: fast peak (6 h), increase with reperfusion; not IVD-R compatible
LPS-binding protein	Increased production if more endotoxins with portal blood to liver	Plasma: LH-tube Amount: 0.1–0.2 mL Method: Human LBP DuoSet ELISA (DY870-05) Provider: R&D Systems (USA)	No data in humans in AMI; not IVD-R compatible
**Tissue ischaemia marker**			
IMA	Ischaemia-marker, not tissue-specific	Serum: CA-tube Amount: 0.1–0.2 ml Method: human IMA ELISA (CSB-E09594h) Provider: Cusabio (China)	Moderate accuracy in humans (4). Animal studies: Fast peak (6 h); not IVD-R compatible
**Inflammatory markers**			
IL-6	Inflammatory marker (not tissue-specific)	Plasma: LH-tube Amount: 0.2 mL Method: Cobas ECLIA; Provider: Roche Diagnostics, Germany) Reference value: < 7 ng/L	Moderate accuracy in humans (4). Animals: fast peak (6 h), increase with reperfusion; IVD-R compatible
PCT	Inflammatory marker (not tissue-specific)	Plasma: LH-tube Amount: 0.2 mL Method: Cobas ECLIA; Provider: Roche Diagnostics, Germany) Reference value: < 0,5 µg/L	Moderate accuracy in humans (4); IVD-R compatible
**Thrombosis/coagulation markers**			
D-dimer	Activation of coagulation (any, not specific to AMI)	Plasma: LH-tube Amount: 0.2 mL Method: Cobas ECLIA; Provider: Roche Diagnostics, Germany) Reference value: < 0.5 mg/L	Moderate accuracy for transmural AMI in humans; IVD-R compatible
SCUBE-1	Activation of coagulation Early marker of thrombosis (any)	Serum: CA-tube Amount: 0.1–0.2 mL Method: Human SCUBE1 ELISA kit (CSB-E15005h) Provider: Cusabio (China)	Not studied in humans. Animals: Peak 6 h? Small increase with reperfusion; not IVD-R compatible
**Other**			
Alpha-GST	A marker of liver injury	Serum: CA-tube Amount: 0.1–0.2 mL Method: Human α-GST ELISA Kit (CSB-E08906h) Provider: Cusabio (China)	Low accuracy in humans (4), no studies on transmural; not IVD-R compatible

*alpha-GST* Alpha- glutathione S-transferase, *CA-tube* Clot activator containing tube with gel, *ECLIA* Electrochemiluminescence immunoassay, *ELISA* Enzyme-linked immunosorbent assay, *IL-6* Interleukin-6, *I-FABP* Intestinal fatty acid-binding protein, *IMA* Ischaemia modified albumin, *IVD-R* In-Vitro Diagnostic Regulation 2017/746, *LH-tube* Lithium-heparin containing plasma tube, *LPS* Lipopolysaccharide, *SCUBE-1* Signal peptide-CUB-EGF domain-containing protein 1

### Data collection

Demographic data, chronic and acute health conditions, times from beginning of symptoms to treatment and diagnosis, details of AMI diagnosis, subtype and management, histology data (if available), and outcome data will be collected (Additional File 1).

### Statistical analysis

#### Sample size

The estimated sample size is in total of 250 patients, 160 patients with confirmed AMI (including 40 patients with NOMI) and 90 patients with suspected but not confirmed AMI.

In the AMESI study, European and West-Asian sites recruited 14 patients with confirmed AMI in average (range 3–34) and 7 (range 0–25) with suspected but eventually not confirmed AMI per site during 10 months. Accordingly, 20 sites are expected to encounter 220 patients with confirmed AMI and 120 patients with suspected AMI during 8 months. Estimating 5% of patients allocated to palliative treatment without any further diagnostics, 10% of referrals and 15% of patients being excluded for other reasons (e.g. missing informed consent, logistical reasons), 20 sites recruiting patients during 8–10 months are necessary to reach the targeted number of patients. As the number of patients with suspected AMI and confirmed NOMI is difficult to predict, the final decision regarding recruitment period will be made after the interim analysis after 4 months of the study.

Statistical analysis will be performed with R Statistical Software 4.3.2 (R Core Team, Vienna, Austria, 2023). Data will be described in number (%), median (interquartile range, IQR) or mean (standard deviation, SD) as appropriate. Kolmogorov–Smirnov test will be used for data distribution normality testing. Analyses for confirmed transmural AMI vs suspected but not confirmed AMI (comparison A – primary outcome), confirmed AMI of any stage vs suspected but not confirmed AMI (comparison B – secondary outcome) and transmural vs non-transmural AMI (comparison C – secondary outcome) will be performed in different measurement time points. Each biomarker will be described at each measurement point for all comparisons (A, B and C), using Student’s t-test or Wilcoxon rank-sum test as appropriate. For all biomarkers showing a *p*-value < 0.05 in at least one measurement point in univariate analysis, the area under the receiver operating curve (ROC) calculation will be performed. We will do this separately for comparisons A, B and C and identify optimal cut-off values for each biomarker based on maximum value of the Youden index [13]. All measurement time points before any treatment of AMI (1,2,3,6,8) will be pooled and AUROC analysis will be performed to identify the best cut-offs also for pooled data for each biomarker. Sensitivity, specificity, positive predictive value (PPV), negative predictive value (NPV) and likelihood ratios (LR) with 95% CI-s will be reported. Positive likelihood ratio (LR +) > 10 and negative likelihood ratio (LR-) < 0.1 will be considered high diagnostic accuracy and LR +  > 5 and LR- < 0.2 as moderate diagnostic accuracy. All biomarkers with at least moderate accuracy will be entered as binary covariates (using the best cut-offs previously found in the AUROC analyses resulting in highest AUC) into the multivariable stepwise logistic regression analysis (both directions) to identify the best combination of biomarkers discriminating between transmural AMI and no AMI based on Akaike information criterion (AIC). Analogical approach will be used for comparisons B and C. The best models will be applied to subgroup analyses in different sub-types of AMI. If the best models selected as described previously, do not perform well at different measurement time points or in different subtypes of AMI, additional analyses will be undertaken testing alternative combinations. We will present the best models for each comparison and for each subtype separately and will use them to construct a practical score that can be used to distinguish between no AMI, non-transmural AMI and transmural AMI (Table 3). If the different cut-offs from different models contradict (for example cut-off for non-transmural AMI vs transmural AMI is lower than the cutoff for no AMI vs AMI) additional analyses will be performed and more appropriate cut-offs will be found considering acceptable sensitivity and specificity from previous AUROC analyses. This approach will be compared to decision tree approach and if the decision tree accuracy is higher than the score’s accuracy, a decision tree will be preferred. The accuracy of the decision tree and the score will be calculated as proportion of correct predictions.

**Table 3:** Hypothetical visualization of a practical score for diagnosis of AMI

	No AMI	Non-transmural AMI	Transmural AMI
Biomarker 1	< x = 0 points	x–y = 1 point	> y = 2 points
Biomarker 2	< x = 0 points		> x = 2 points
Biomarker 3	< x = 0 points	x–y = 1 point	> y = 2 points
Biomarker 4	< x = 0 points	> x = 1 point	
Biomarker 5	< x = 0 points		> x = 2 points

This visualization is hypothetical. PPV – positive predictive value

Hypothetical examples: 0–1 points = no AMI (PPV; 2–4 points = non-transmural AMI (PPV); > 5 points = transmural AMI (PPV)

Dynamics of each biomarker will be visualized graphically in patients with confirmed AMI differentiating between patients in whom initial treatment was successful from patients in whom bowel ischaemia was ongoing after initial treatment, with the aim to explore possible differences in biomarker trajectories in case of reperfusion and ongoing bowel ischaemia.

### Ethics

Primary ethical approval has been obtained from the Research Ethics Committee of the University of Tartu (Approval No. 386/T-11). Each participating site will apply for local Ethics Committee approval according to country and institutional regulations.

The first blood sampling should be performed immediately after arrival to the hospital or at suspicion of AMI to allow appropriate analyses. Several biomarkers are expected to peak already within 6 h of the ischaemic event. Accordingly, the first sampling needs to take place immediately, before informed consent with proper explanation of the study can be obtained from patients usually presenting in severe condition. Study sites are encouraged to apply for delayed informed consent that can be obtained from the patient or patient’s next of kin/proxy at the first possibility after initiating sampling and data collection.

If next of kin has given the primary consent, the patient’s consent will be sought as soon as they regain the ability to understand and give informed consent. Patients will be excluded from the study and any samples taken will be discarded and data already collected deleted if the patient or the patient’s next of kin declines participation in the study.

Data will be recorded in an electronic Case Report Form in a pseudonymized way. Each patient receives an identification number at the site; no personal data allowing identification of the patient are included in the database. Patients are identifiable only at the site via patient log, accessible only to the primary investigator at each site.

The electronic Case Report Form will be created using the REDCap platform and stored on a secure server of the University of Tartu. Investigators will have access to their own local data, to enter and edit on the database, until all data collection is complete. Only the PI, the central study team in Estonia and the data quality control and analysis team will have access to the full database.

The maximum amount of blood drawn from one patient is 96 mL (less for most patients depending on clinical scenario), being well within safe limits for adult patients [14]. If necessary, according to each respective site´s national regulations, there will be an application to the local biobank for agreement on storage of plasma samples in a freezer of -80° C for shipment of frozen samples at four months after study start and at end of study to central laboratory at Tartu University Hospital.

### Compliance with reporting guidelines and methodological literature

The protocol has been reported according to the Strengthening the Reporting of Observational Studies in Epidemiology statement (Additional file 2) [15] and Standards for the Reporting of Diagnostic Accuracy Studies guidelines (Additional file 3) [16].

## Discussion

Research on AMI has been hampered by the relatively rare occurrence and difficulties in diagnosis. Patients are encountered by different specialties and collaboration to identify all cases throughout the whole hospital is difficult. However, recent multicentre study confirmed that such effort is possible and may reveal important differences between the hospitals [3]. Studies on biomarkers have important limitations [4]. In our opinion, disappointing results from studies to date do not allow definitive conclusion about availability of accurate biomarkers. The ongoing study in the Netherlands [17] is the first ever assessing serially measured biomarkers in a multicentre study. This is undoubtedly an important study, certainly making an important contribution to knowledge, however, several potential biomarkers are not considered.

The current study will be multicentre, including patients with both, suspicion of AMI and confirmed AMI, assessing potential combinations of biomarkers and aiming to find biomarkers for early diagnosis of AMI as well as differentiating transmural bowel necrosis from non-transmural, thus helping to guide important treatment decisions. The study will be an effort for all sites from the practical point of view, to include patients from different parts of the hospital and manage sampling and sample processing at any time of the day. Real recruitment cannot be well predicted, and sample size precisely calculated. Laboratory analyses will be challenging due to logistics and workload. Measurement kits for some novel biomarkers are not fully validated and measurement and interpretation issues may occur. A major strength, however, is the use of one assay per biomarker in one core lab. Statistical analysis is highly complex, considering several measurement points, different severity and different sub-types and the aim to have a combination of biomarkers. Subgroups analyses based on sub-types of AMI will be expectedly limited by relatively small number of patients with different clinical scenarios. Some biomarkers are expected to overlap with specific clinical scenarios (e.g. troponin dynamics in a patient with NOMI due to cardiogenic shock after myocardial infarction vs. in a patient having myocardial injury secondary to AMI). Additionally, several other biomarkers may reflect severity of illness (e.g. acidosis, creatinine) rather than directly describing AMI, potentially complicating interpretation of results. The risk that a combination of biomarkers allowing accurate prediction of AMI or transmural AMI will not be identified in this study is considerable.

Despite all these challenges we are convinced that it is possible and clearly necessary to conduct this study and that it will result in a relevant contribution to our knowledge. We invite anyone interested in the study to contact us to be considered for involvement in this international study.

### Supplementary Information

Supplementary Material 1.

Supplementary Material 2.

Supplementary Material 3.

## Data Availability

No datasets were generated or analysed during the current study.
